# Supplementary material for: The interactome of intact mitochondria by cross-linking mass spectrometry provides evidence for coexisting respiratory supercomplexes
Source: Mol Cell Proteomics. 2017 Dec 8;17(2):216–32. doi: 10.1074/mcp.RA117.000470 (PMC5795388; doi:10.1074/mcp.RA117.000470)
Supplement: Supplemental Data [file supp_RA117.000470_133922_0_supp_23978_fzffwf.zip › spectra_annotation/mito_DR_spectra_annotation/422-1-3-1-1-1.pdf]

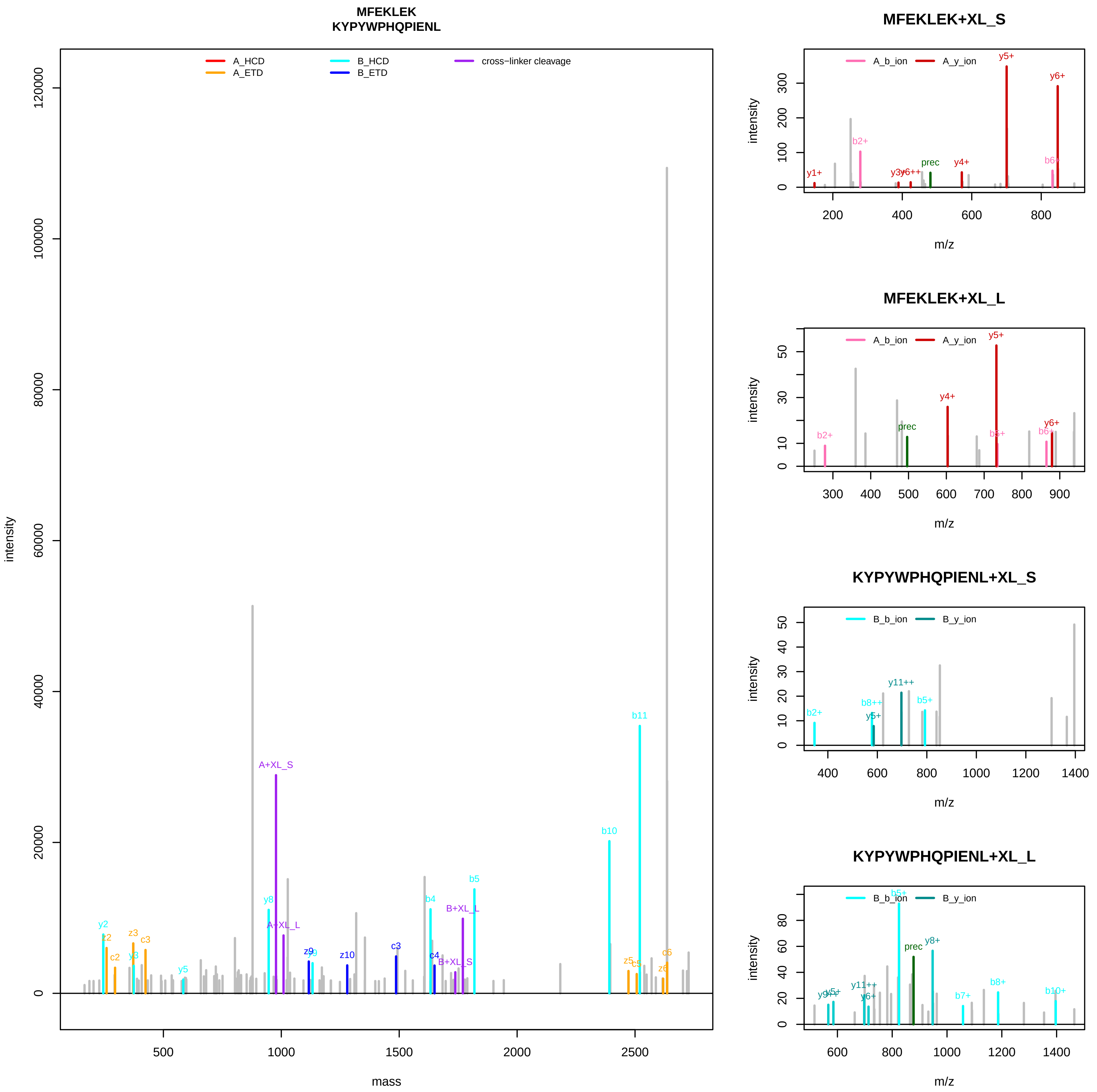

MFEKLEK
KYPYWPHQPIENL
A_HCD
A_ETD
B_HCD
B_ETD
cross−linker cleavage
intensity
mass
A+XL_S
A+XL_L
B+XL_L
B+XL_S
b11
b10
b5
b4
y8
y2
y3
y5
y9
z2
z3
c2
c3
z9
z10
c3
c4
z5
c5
z6
c6
MFEKLEK+XL_S
A_b_ion
A_y_ion
m/z
MFEKLEK+XL_L
KYPYWPHQPIENL+XL_S
B_b_ion
B_y_ion
KYPYWPHQPIENL+XL_L
prec
